# Immune profile and immunosurveillance in treatment-naive and neoadjuvantly treated esophageal adenocarcinoma

**DOI:** 10.1007/s00262-019-02475-w

**Published:** 2020-01-20

**Authors:** Svenja Wagener-Ryczek, Max Schoemmel, Max Kraemer, Christiane Bruns, Wolfgang Schroeder, Thomas Zander, Florian Gebauer, Hakan Alakus, Sabine Merkelbach-Bruse, Reinhard Buettner, Heike Loeser, Martin Thelen, Hans A. Schlößer, Alexander Quaas

**Affiliations:** 1 Department of General, Visceral, Cancer and Transplantation Surgery, University Hospital Cologne, Cologne, Germany; 2 Institute of Pathology, University Hospital Cologne, Cologne, Germany; 3 Department I of Internal Medicine, Center for Integrated Oncology (CIO), University Hospital Cologne, Cologne, Germany; 4 Center for Molecular Medicine (CMMC), Cologne, Germany; 5 Gastrointestinal Cancer Group Cologne (GCGC), Cologne, Germany

**Keywords:** Esophageal adenocarcinoma, Immune profile, RNA expression, Nanostring

## Abstract

The outcome in esophageal adenocarcinoma (EAC) is still poor with only 20% of patients in Western populations surviving for more than 5 years. Almost nothing is known about the precise composition of immune cells and their gene expression profiles in primary resected EACs and also nothing compared to neoadjuvant treated EACs. This study analyzes and compares immune profiles of primary resected and neoadjuvant treated esophageal adenocarcinoma and unravels possible targets for immunotherapy. We analyzed 47 EAC in total considering a set of 30 primary treatment-naive EACs and 17 neoadjuvant pretreated (12 × CROSS, 5 × FLOT) using the Nanostring's panel-based gene expression platform including 770 genes being important in malignant tumors and their immune micromileu. Most of the significantly altered genes are involved in the regulation of immune responses, T-and B cell functions as well as antigen processing. Chemokine-receptor axes like the CXCL9, -10,-11/CXCR3- are prominent in esophageal adenocarcinoma with a fold change of up to 9.5 promoting cancer cell proliferation and metastasis. ARG1, as a regulator of T-cell fate is sixfold down-regulated in untreated primary esophageal tumors. The influence of the currently used neoadjuvant treatment revealed a down-regulation of nearly all important checkpoint markers and inflammatory related genes in the local microenvironment. We found a higher expression of checkpoint markers like LAG3, TIM3, CTLA4 and CD276 in comparison to PD-L1/PD-1 supporting clinical trials analyzing the efficacy of a combination of different checkpoint inhibitors in EACs. We found an up-regulation of CD38 or LILRB1 as examples of additional immune escape mechanism.

**Electronic supplementary material:**

The online version of this article (10.1007/s00262-019-02475-w) contains supplementary material, which is available to authorized users.

## Introduction

Esophageal adenocarcinoma (EAC) is associated with the sixth-highest cancer-related mortality and increasing incidences mainly in the Western World [1, 2]. Curative treatment consists mostly of a multimodal therapy of esophageal en-bloc resection and perioperative radio-chemotherapy, but compared to other cancer entities the outcome is still poor with only 20% of patients in Western populations surviving for more than 5 years [3–5].

There is a high need for new therapeutic approaches in treating this cancer [6].

The interaction of tumor cells and associated immune compartment is supposed to play an important role in cancer progression. Mechanisms of immunosuppression within the tumor and its microenvironment are incompletely understood, although neoantigen loss and negative regulation by immune checkpoints are presumed to lead to dysfunction of specialized T-cells [7, 8].

Immune checkpoint inhibitors (e.g. Pembrolizimab, Nivolumab) enhancing antitumor T-Cell activity through the inhibition of immune checkpoints, like the programmed death-1 (PD-1) receptor [9] and improved survival in some solid tumors like malignant melanoma and non-small cell lung carcinoma [10–13]. First line and second line treatment of metastatic esophageal cancer with checkpoint inhibitors considering the PD1/PDL1 axis are currently tested in a Phase III evaluation with pembrolizumab (KEYNOTE-062, KEYNOTE-061) [14] as well as nivolumab (CheckMate-577) in the adjuvant setting with various other approaches in all lines of therapy [15].

Almost nothing is known about the precise composition of immune cells and their gene expression profiles in primary resected EACs and also nothing compared to neoadjuvant treated EACs.

Due to the fact that most EACs are neoadjuvantly treated, the question arises as to what effects neoadjuvant treatment has on the local immune micromileu in carcinoma?

Accordingly one aim of our study was to analyze and compare the immune profile of primary resected as well as neoadjuvant treated esophageal adenocarcinoma and to unravel possible targets for immunotherapy as for example cancer testis antigens (CTA) that have been shown to exhibit characteristics important for tumorigenesis. Targeting such antigens may control cancer progression [16]. Additionally we compared the primary resected EACs in their regulation of genes known to be associated with response to PD-1/PD-L1 inhibitors. This so-called hot inflammation profile consists of 18 genes associated with a T cell-inflamed and IFN-γ-related response to antigen presentation, chemokine expression, cytotoxic activity, and adaptive immune resistance [17].

To address this question, we used the NanoString technology. Nanostring's panel-based gene expression platform, in particular, considers 770 genes that have been described as important in malignant tumors and their immune micromileu.

## Materials and methods

### Clinical characteristics of study cohort

We analyzed formalin-fixed and paraffin embedded material of 47 patients with esophageal adenocarcinomas (EAC). More than 80% (*n* = 40) had locally advanced stages of EAC (T2 or more) and were predominantely men (89%) between 45 and 85 years old at the date of surgery.

Thirty patients (64%) received primary surgical resection (without neoadjuvant treatment) between 2014–2017 at the Department of General, Visceral and Cancer Surgery, University of Cologne, Germany. Standard surgical procedure was laparotomic or laparoscopic gastrolysis and right transthoracic en-bloc esophagectomy with intrathoracic esophagogastrostomy including two-field lymphadenectomy of mediastinal and abdominal lymph nodes or transhiatal extended distal esophagectomy with transabdominal intrathoracic or cervical anastomosis as described previously described [18].

Seventeen patients (46%) had received neoadjuvant (radio)-chemotherapy. Four patients received chemotherapy alone according to the FLOT protocol (docetaxel, oxaliplatin, fluorouracil/leucovorin), one patient according to the ECX protocol (epirubicin, cisplatin, capecitabine) and 12 patients a combined radio-chemotherapy according to the CROSS protocol (paclitaxel, carboplatin and 41.4 Gy/23 fractions). We considered patients with at least 50% remaining tumor tissue after neoadjuvant treatment. High quality RNA was extracted from tumor of all 47 patients for NanoString Analysis. Accordingly, RNA from tumor-free tissue of 10 patients in the primary naive tumor cohort was extracted as healthy normal control.

### Macrodissection and RNA isolation

All samples were routinely formalin-fixed and paraffin embedded (FFPE) according to local practice. Histological specimens were evaluated by board certified pathologists. 10 μm thick sections were cut from FFPE tissue block for RNA extraction. Six sections of 10 μm thickness were deparaffinized and the tumor areas were macrodissected from unstained slides using a marked hematoxylin–eosin (H&E) stained slide as a reference. For extraction the Maxwell RSC RNA FFPE Kit was used on the Maxwell RSC (Promega) according to manufacturer’s instruction, including DNAse digestion.

### Expression analysis

Differential expression of immune related genes on mRNA level was determined using the NanoString PanCancer Immune Profiling Panel (NanoString Technologies, Inc., Seattle, WA). Isolated RNA was hybridised to a set of 770 specific and fluorescently labelled gene probes for 18 h @ 65 °C. Afterwards hybridisation products were prepared for cartridge loading on an nCounter PrepStation. Digital Counting of fluorescent signals was conducted using the nCounter Digital Analyzer. Afterwards data analysis including statistics was carried out with the nsolver3.0 software and the advanced analysis 2.0 package. 40 houskeeping genes within the panel facilitated sample-to-sample normalization.

## Results

### Immune profile in primary treatment-naive esophageal adenocarcinomas

We first analyzed a set of 30 primary treatment-naive EACs and compared these gene expression results to matched normal esophageal mucosa (Table 1). Most of the significantly altered genes are involved in the regulation of immune responses, T-and B cell functions as well as antigen processing (Sup. Figure 1 and 2).

**Table 1:** Patients’ characteristics

Neoadj.therapy	Naive		CROSS		FLOT		Total	
*n* = 47 (Eurasian)	*n*	%	*n*	%	*n*	%	*n*	%
	30	63,8	12	25,5	5	10,6	47	100
Sex								
M	26	55,3	11	23,4	4	8,5	41	87,2
F	4	8,5	1	2,1	1	2,1	6	12,8
Age								
> 50	29	61,7	9	19,1	5	10,6	43	91,5
< 50	1	2,1	3	6,4	0	0	4	8,5
T-stad								
(y)T1/2	10	21,3	4	8,5	1	2,1	15	31,9
(y)T3/4	20	42,6	8	17	4	8,5	32	68,1
N stage								
pN0	16	53.3	5	41.6	1	20	22	46.9
pN1	12	40.0	5	41.6	3	60	20	42.6
pN2	2	7.0	2	16.8	1	20	5	10.5
Number of pos lymphnodes	3.4 (0–21)		2.2 (0–12)		6,4 (0–14)		3.4 (0–21)	
Number of resected lymphnodes	30.9 (18–51)		29 (18–51)		34 (12–52)		30.8 (12–52)	
Tumor length	2.2 (1.0–4.5)		2,1 (1.0–4,1)		1,9 (0.9–3.9)			
Smoking								
Yes	13	43.3	1	9,1	0	0	14	
No	13	43.3	7	54,4	5	100	25	
Former smoker	4	13.3	4	36,5	0	0	8	

### Factors that interfere with the antigen processing machinery

**Fig. 1:**
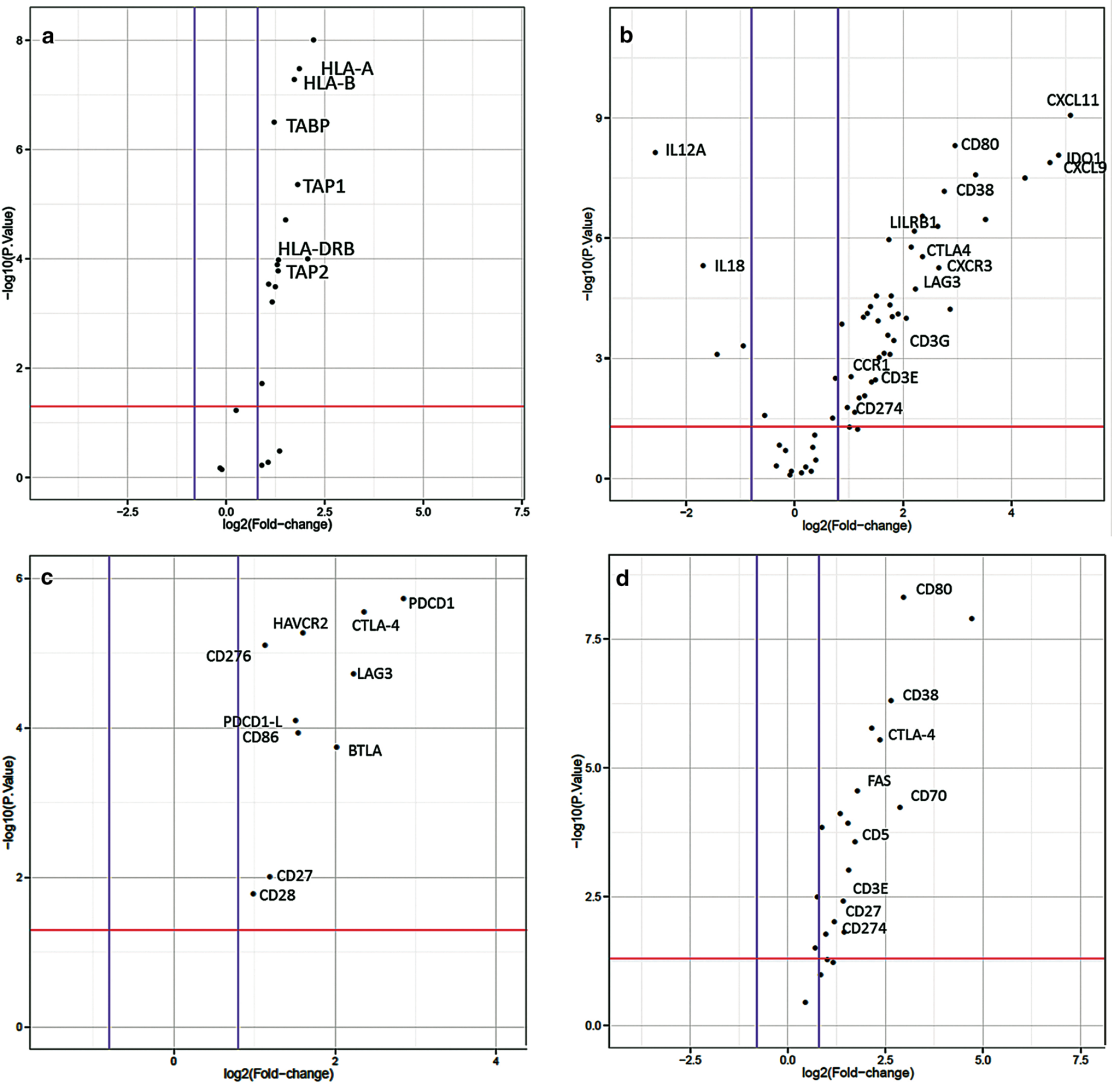
Differential expression of immune-related genes.  Upregulated expression of genes related to **a** antigen presentation. ‘Volcano plot’ of statistical significance against fold-change between primary EAC and normal tissue, demonstrating the significantly differentially expressed genes of MHC class I and II. **b** Expression of genes related to T-cell function. ‘Volcano plot’ of statistical significance against fold-change between primary EAC and normal tissue, demonstrating the significantly differentially expressed genes, CD3E, CD3G, LAG3, CTLA-4, LILRB1, CD38. **c** Expression of checkpoint genes. ‘Volcano plot’ of statistical significance against fold-change between primary EAC and normal tissue, demonstrating the significantly differentially expression of PDCD1, CTLA-4, HAVCR2, CD276, LAG3, PDCD1-L1, CD86, BTLA, CD27 and CD28. **d** Expression of genes related to B-cell function. ‘Volcano plot’ of statistical significance against fold-change between primary EAC and normal tissue, demonstrating the significantly differential expression of B-cell function related genes. Thresholds of significance *p*-value:1.3 (red line); log2FC: 0.8 (blue lines)

Within the whole study population of primary and NACT treated tumors (see Table 1 for patients´ characteristics), expression of MHC class I and II genes was upregulated in EAC compared to normal tissue (Fig. 1a). Nevertheless, we asked whether inhibition of macrophage phagocytosis by MHC class I could be altered in esophageal tumor specimen by an alternative way to interfere with antigen processing. We thereby identified LILRB1 to be significantly upregulated (fold change 2, *p* = 0.0005). LILRB1 plays a major role as a receptor in the detection and simultaneous inhibition of MHC class I triggered phagocytosis (Fig. 1b).

### T-cell status

In the group of therapy-naive tumors, the following deviations from the normal sample should be emphasized:

CTLA-4 expression is significantly increased in primary resected, treatment naive EAC (*p* = 0.01) (Fig. 1c) as well as the costimulatory molecules CD80 and CD86 (Fig. 1c, d), which trigger higher interaction with CTLA-4. Further co-stimulatory and checkpoint molecules that are significantly overexpressed are CD70, which interacts with CD27 and TIM-3 (HAVCR2), which is also known as an important checkpoint molecule (Fig. 1c). Noteworthy also CD38 is overexpressed in primary esophageal tumors and represents a known mechanism of resistance to PD-1/PD-L1 blockade (Fig. 1b).

### T-cells in treatment-naive- and post- radiochemotherapy (NACT) samples

T cell enrichment as measured by CD3 expression was found to be upregulated in treatment naive EACs by a factor of three in comparison to normal tissue (Fig. 1b). In contrast, post-NACT-treatmed samples presented a decrease in CD3 expression in comparison to treatment-naive samples, but present still a twofold normal tissue expression.

**Fig. 2:**
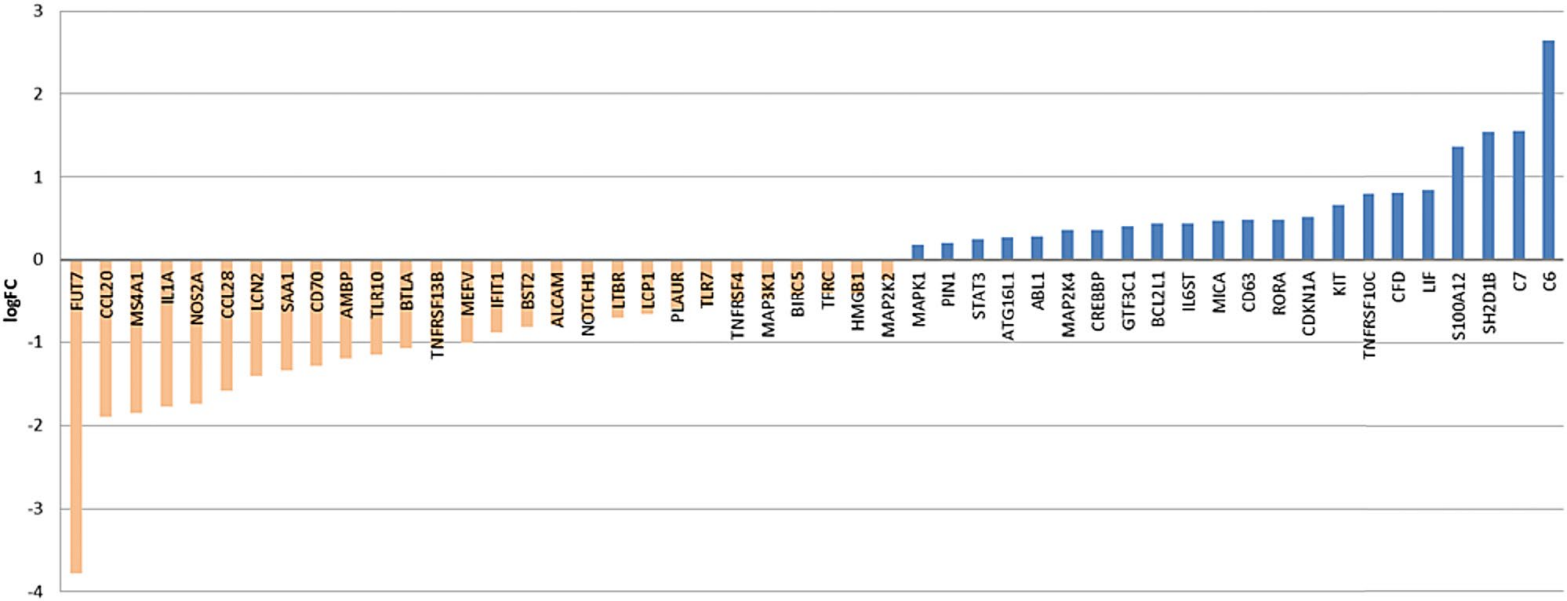
Top 50 differentially expressed genes post NACT vs primary EAC. Waterfall plot for log2 fold-changes in mRNA gene expression levels in post NACTvs primary resected, treatment- naive EAC. Significantly downregulated genes are marked in orange and upregulated genes are marked in blue

Expression of the T-cell co-receptor CD8, which binds the MHC-I on antigen presenting cells, is increased threefold in tumor tissue regardless of therapy. CD8a expression varies between individual patient samples, nevertheless they all show a higher expression compared to normal tissue. CD8b receptor chain expression had a higher intra-group variability with some specimen showing expression values closely to normal expression. NACT was determined not to influence expression of CD8a and CD8b (expression of both CD8 chains alpha and beta was determined to remain stable under NACT). Within the top 50 genes, determined to be differentially expressed post NACT versus primary treatment-naive EAC, none of the above mentioned genes are present (Fig. 2 and Sup Fig. 3).

### Factors that interfere with the function of activated T cells

#### Expression of different immune checkpoints (CTLA-4, CD-276, TIM-3, LAG-3, PD-1 and PDL-1).

In the following we evaluate differences in expression between therapy-naive tumors compared to the normal tissue and to neoadjuvant pretreated tumors (NACT): As already indicated, CTLA-4 expression is 3.5–4fold increased in esophageal tumor specimen compared to normal tissue (Fig. 1c). We observed a low variability of CTLA-4 expression between different patient samples, except one tumor with a very high and one sample with a very low expression of CTLA-4. The immune checkpoint receptor CTLA-4 plays a crucial role in the negative regulation of T-cell activation of cancer cells to evade immune response and maintain self-tolerance. The PD-L1 expression in all (primary untreated as well as NACT)—tumor specimens investigated is only slightly increased compared to normal tissue (Fig. 1c). NACT treatment per se does not seem to have an effect on PD-L1 status. PD-1 shows a 2–3 fold expression in comparison to normal healthy tissue (Fig. 1c). Intra-group variation was astonishingly low except few outliers, known that PD-1 expression is heterogeneous in other cancer entities. PD-1 expression status does not seem to be different in NACT treated samples.

Besides CTLA-4 and PD-1/PD-L1, there are further upcoming immune checkpoints gaining attention in preclinical trials for specific blocking antibodies (e.g. LAG3, TIM-3).

HAVCR2 (TIM-3) exhibits a threefold increased expression in esophageal tumor samples compared to normal control samples (Fig. 1c). Further, we evaluated whether TIM-3 expression is influenced by NACT and found no differential expression between both patient groups. LAG3 shows a fourfold higher expression in primary untreated esophageal tumor in comparison to healthy normal tissue (Fig. 1c). In contrast to HAVCR2, LAG3 expression is strongly reduced by the factor of two in the neoadjuvant treated group.

CD-276 (also known as B7-H3) showed a significantly increased expression in primary naive esophageal tumors as well. CD276 belongs to PD1-checkpoint family and elicits a similar inhibitory effect on T-cells as PD-1 does (Fig. 1c).

#### Secretion of immuno-modulating molecules

Noteworthy are in particular two markers (ARG1 and IDO1) that cooperate to establish an immunosuppressive tumor microenvironment. Both showed significant differences in expression to the normal tissue as well as to the neoadjuvant treated group:

ARG1, as a regulator of T-cell fate is sixfold downregulated in untreated primary esophageal tumors and fourfold downregulated in the NACT group in comparison to normal tissue.

IDO-1, which induces tolerance to self-antigens via inhibition of T cell activation, exhibits a sevenfold higher expression in primary untreated esophageal tumor in comparison to healthy normal tissue (Fig. 1b), whereas it has a fourfold higher expression in NACT treated patients. Thus representing a twofold downregulation of IDO-1 by NACT therapy. Nevertheless there is a high intra-group variability of IDO-1 expression in different patients with 2–3 fold higher expression values.

### Factors that interfere with homing of activated T cells

Immune cells are regulated by many different chemokines. Therefore, we thought to investigate, which chemokine-receptor axes are prominent in esophageal adenocarcinoma samples. Interestingly we found the CXCL9, -10,-11/CXCR3 (fold change 9.5, 8, 14 and 2.5 with *p* = 0.0005–0.0001) axis to be significantly upregulated in the tumor tissue, promoting cancer cell proliferation and metastasis (autocrine axis) (Fig. 1b).

### Expression of a Tumor inflammation signature that predicts response to immunotherapy

**Fig. 3:**
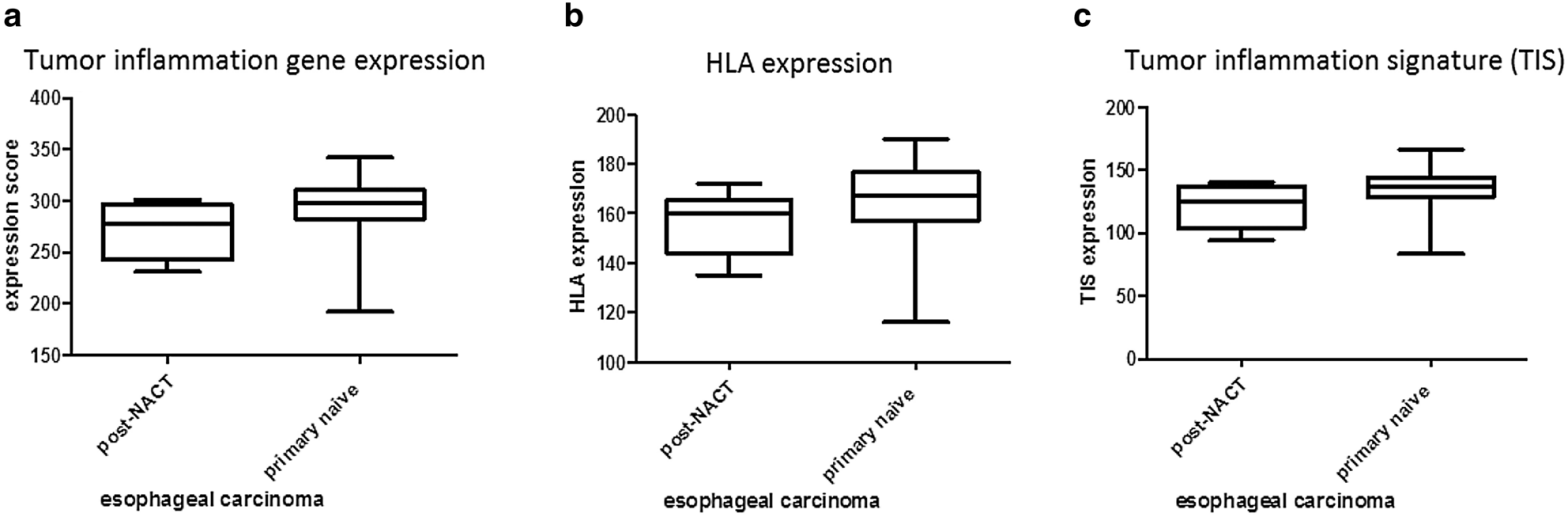
Scored expression of HLA genes and tumor inflammation markers. Expression score of primary naive EAC and post NACT of combined HLA- and TIS expression (**a**), HLA expression (**b**) and tumor inflammation signature (TIS) as defined by Merck (**c**)

**Fig. 4:**
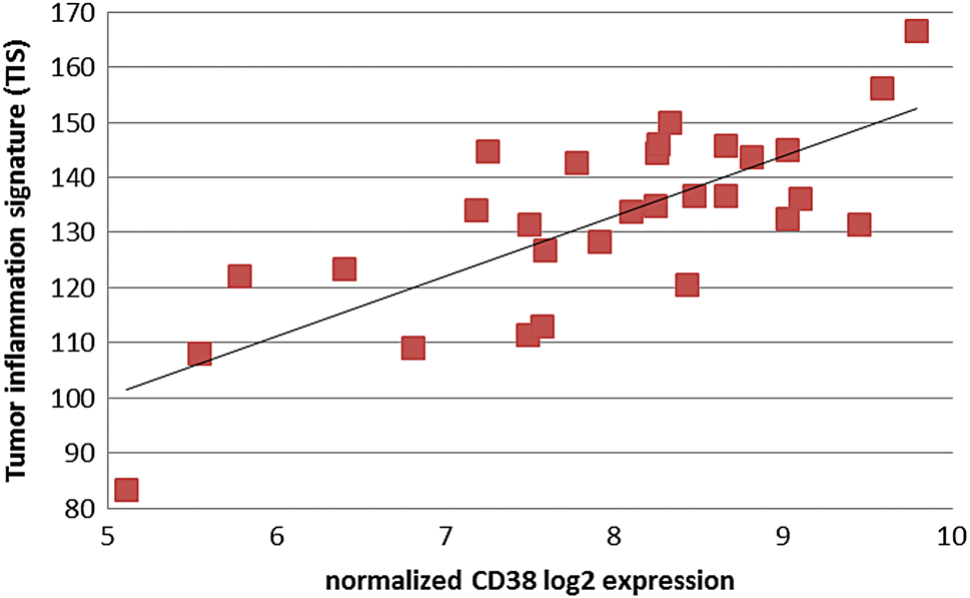
Correlation of CD38 expression with the Tumor Inflammation Signature (TIS). Correlation coefficient *r* = 0.86 indicates a strong correlation between CD38 expression and an inflammatory tumor phenotype as defined by the tumor inflammation signature (TIS)

Primary untreated and neoadjuvant treated esophageal adenocarcinoma specimen were analyzed for their tumor inflammation signature, as developed by Merck and Nanostring to predict response to PD-1 blockade [19]. Afterwards samples were scored according to the sum of inflammation signature gene expression. Comparison of primary untreated to neoadjuvant treated esophageal adenocarcinoma specimen revealed HLA—and cancer testis antigens (CTA) expression to be comparable in both groups (Fig. 3a, b). Nevertheless, therapy-naive esophageal tumor specimen showed a numerical higher inflammation signatures as well as HLA expression (Fig. 3c). As described above, we could show a high CD38 expression within primary EAC. To unravel possible combinatorial treatments we determined whether CD38 expression correlates with the tumor inflammation signature (TIS) (Fig. 4). We analysed the probability and duration of survival for primary-resected and neoadjuvantly treated EAC patients as shown in the Kaplan-Meyer Curves (Sup. Figure 4). Regarding the patients´ survival, there is a statistically significant difference between primary resected and the NACT-treated subgroup. A high inflammatory phenotype (primary EAC) correlates with a higher probability of survival, whereas NACT-treated tumors with a downregulated immune response, show a reduce probability of survival (46 vs. 32 months).

### Differences in immune related gene expression upon different perioperative therapy protocols (FLOT vs. CROSS)

**Fig. 5:**
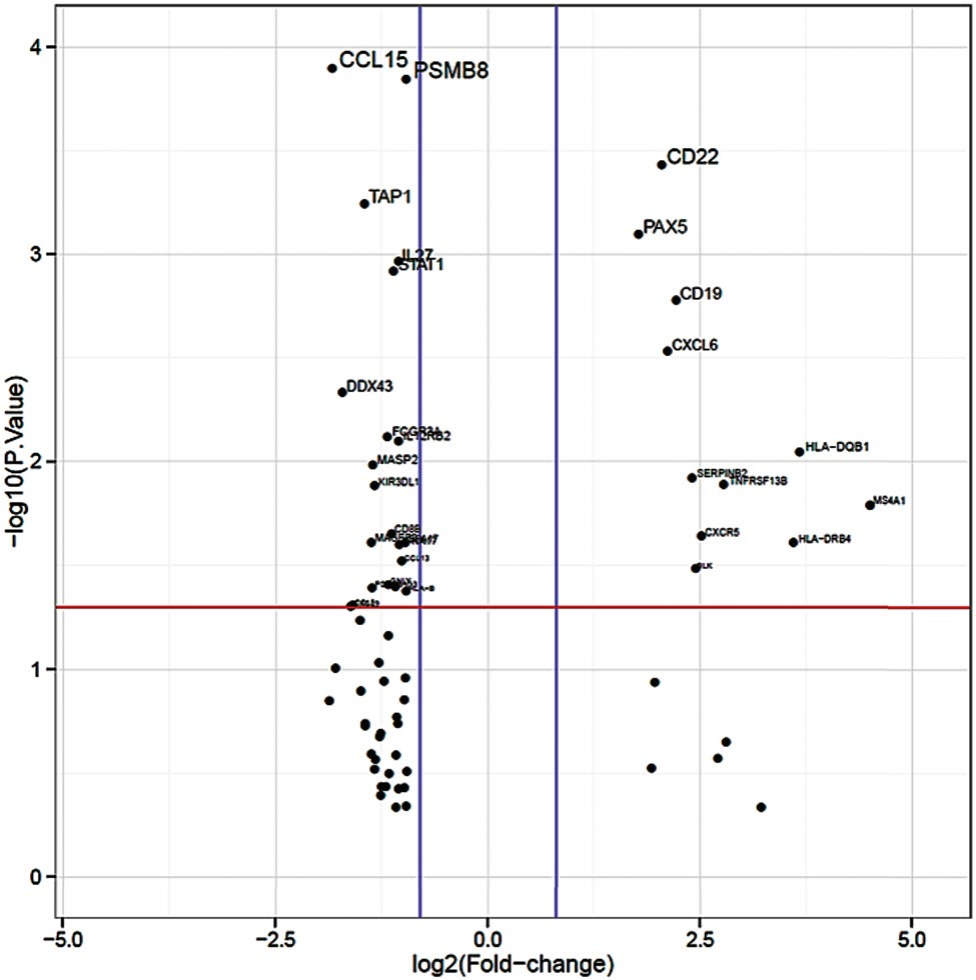
Differentially expressed genes of FLOT-vs CROSS protocol treated patients. ‘Volcano plot’ of statistical significance against fold-change between post-NACT treated patients with FLOT vs CROSS protocol, demonstrating the significantly differentially expressed genes. Thresholds of significance *p*-value 1.3 (red line); log2FC: 0.8 (blue lines)

We further asked, whether the type of neoadjuvant treatment protocol might have influenced the expression of central immune response related genes. Patient samples neoadjuvant pretreated with chemotherapy (FLOT) showed significantly upregulated gene expression of MHC class II molecules in comparison to neoadjuvant pretreated patients according to the CROSS protocol (radio-chemotherapy). Furthermore important factors of B cell activation (B cell receptor signaling) are significantly upregulated like the B cell receptor component CD79A, CD79B or its associated co-receptor genes CD19 and C21. In contrast we observed a downregulation of MHC class I genes and the MHC I associated TAP transporter genes TAP1 and TAP2 (Fig. 5).

## Discussion

Conventional oncological treatment regimens such as chemotherapy or radiotherapy are inadequate effective in EACs. Personalized therapy options are limited to HER2 blockage for a limited patient group showing a median advantage in progression-free survival of less than 3 months. Further therapy options are urgently needed.

Currently checkpoint-inhibitors like pembrolizumab and nivolumab which have proven to be effective, inter alia, in the treatment of malignant melanoma and NSCLCs are tested in different Phase III for esophageal cancer. First preliminary study results for pembrolizumab as a second-line treatment demonstrated an improved overall survival (OS) in patients with advanced or metastatic esophageal or esophagogastric junction carcinoma [20].

The structure of the studies available to date illustrates a significant problem. The studies mimic gastric adenocarcinomas with the adenocarcinomas of the esophagus (and subsume these as adenocarcinomas of the gastroesophageal junction) in the erroneous assumption that there are no relevant differences in tumor biology. As a matter of fact gastric adenocarcinomas reveal just for immunotherapy relevant subgroups such as microsatellite-instability (MSI) and Epstein–Barr-virus-related (EBV) subgroup, which are exceedingly rare or missing in adenocarcinomas of the esophagus [21, 22].

We have therefore focused on adenocarcinomas of the esophagus in this study.

The immune system interacts with esophageal adenocarcinomas in many ways and thereby substantially affects tumor progression and therapeutic response. Nearly nothing is known about these important interactions in EAC. Consequently the main focus of our study was to unravel the immune profile of EAC as defined by their T-cell activity, inflammation signature and immune escape mechanisms. Although most conventional therapies can elicit immune responses contributing to their efficacy, we could also show that radio-chemo therapy negatively alters the local immune status.

Other studies already identified molecular subtypes linked to the clinical outcome after immunotherapy. For example, different molecular subtypes have been identified in colorectal cancers which define potential strategies for immunotherapy [23]. Multiple characteristics are proposed to be responsible for a certain immune microenvironment as well as related mechanisms of immune escape. The consensus molecular subtype I (CSM I) for colorectal carcinoma is characterized by a high expression of PD-1, CTLA-4, IDO1 and other immune checkpoints. Moreover, its immune regulation is mainly driven by the chemokine CXCR3/CCR5 axis and cytotoxic effector mechanisms that are critical for activation and differentiation of T cells. CSM type IV in contrast is definied by an increased TGF-b signaling and upregulated CXCL-12, which drive inflammation and metastasis formation. Upon our findings, that primary untreated patients with EACs showed a high expression of major immune checkpoints as well as an upregulated CXCR3/CCR5 axis, it would be interesting to define prognostic phenotypes and thereby directing therapeutic strategies. In addition to this, we identified a subgroup of EAC patients with ultra-high expression of cancer testis antigens (CTAs), which displayed a significant upregulation of genes associated with tumor progression and metastasis formation. We therefore suspect, the score of CTAs to be a possible prognostic marker for clinical outcome in EAC as already identified for other tumor entities [16].

So far, neoadjuvant radiochemotherapy (RCT) is a well-established first-line treatment in patients with esophageal cancer. Nevertheless we here observed a significant decrease of T cell activity as measured by CD3 and CD8 expression after RCT. This finding implies that RCT impairs lymphocyte activity as well as components of the adaptive immune response, as targets of immunotherapy. Since the composition of the tumor microenvironment with immune cells and chemokines mainly drives efficacy of immunotherapy [24] and RCT profoundly suppresses the adaptive immune response, we propose that a combination of both could be restricted. Similar observations have been made in cervical and colorectal cancer patients [25, 26].

Restricting, however, we must state at this point that this explanation refers only to the local tumor micromileu. Memory cells in surrounding lymph nodes could trigger an effective neoantigen-driven tumor cell-destroying inflammatory response regardless of the local situation. Further it has to be kept in mind that the patient cohort of NACT-treated EAC includes only a small sample size. Nevertheless, these results can be an argument for clinical trials considering the use of checkpoint inhibitors first-line. We therefore propose to further validate the above described findings in future studies.

Controversely, we could not observe any upregulation of PD-1 expression upon chemotherapy as described previously [25, 26]. This might be due to the fact, that primary esophageal tumor samples show no differential expression of PD-1 and PD-L1 at all. This evidence further suggests that PD-1 blocking agents, which have shown to be promising in NSCLC and renal cancer as well as melanoma, might not be as effective in esophageal adenocarcinoma or at least just in a small subset of patients with EACs. Nevertheless, recent clinical trials reveal efficacy of checkpoint inhibitors also in PD-L1 low expressing patients. This phenomenon is currently investigated [27] and noteworthy within our study, other checkpoint molecules like HAVCR2 (TIM-3), LAG-3 and CTLA-4 are dominantly expressed and therefore promising therapy markers/targets (see discussion below). Furthermore most EACs show a high mutational burden (TMB) which is correlated with good clinical response to checkpoint inhibition in NSCLC. Interestingly, a family member of PD-1, CD276 that even elicits similar inhibitory effects on T-cells is dominantly upregulated in primary EAC. Recently, CD276, also known as B7-H3, was identified to decrease levels of IFN-y, TNF alpha and inflammatory cytokines and thereby allowing immune escape [28].

Tumor escape from anti-tumor immunity is a critical event for tumor survival and progression [29]. Different mechanisms have been described and discussed extensively in the past [30, 31]. These include loss of antigenicity by modulation of the antigen presenting machinery. Downregulation of the antigen presenting MHC- class 1 has been found in various solid malignancies like melanoma, lung, breast and prostate cancers [32]. Primary EAC samples within our study cohort display increased MHC class I expression on mRNA level compared to normal tissue. In contrast IHC screening identified approximately 30% of EAC to have a loss of MHC marker expression on their tumor cell surface. Nevertheless we could identify other inhibitors of MHC class I-linked macrophage phagocytosis on mRNA expression level. Interestingly the major receptor in detection and simultaneous inhibition of MHC class I triggered phagocytosis, LILRB1, was significantly upregulated and could explain a possible tumor escape mechanism [33]. Further, tumors, which retain sufficient antigen presentation for immune recognition can still escape from elimination by downregulation of their immunogenicity, for example by the expression of immuno-inhibitory molecules (receptors and ligands) like PD-1/PD-L1, LAG3 and HAVCR2 (TIM-3) [30]). Also the microenvironment with infiltrating tumor lymphocytes (TILs) and T cell suppressing enzymes enhances immunoresistance. The ability of tumors to orchestrate this surrounding environment determines the cellular fate of TILs and allows evasion from immune elimination [34]. Enhancing efficacy of immunotherapy needs to consider immune escape mechanisms by immune profiling. Interestingly, within our cohort of primary naive esophageal carcinoma, distinct immune escape mechanisms are dominant, while others are not present. In detail, our cohort of primary EAC showed an upregulation of checkpoint inhibitors as most prominent mechanism of immune evasion with 7–4 fold increased expression of CTLA-4, HAVCR2 (TIM-3) and LAG3. Modulation of the tumor microenvironment as an enhancement of immunosuppression is prominent within primary naive EAC. We could identify high tumor inflammation signatures within nearly all patient samples compared to normal tissues. Approximately 50% of the samples elicit an extremely high score of inflammation markers. Furthermore, we could show a high CD38 expression within primary EAC, which was recently determined to be influenced by CD8 + T-cells within the tumor microenviroment and consequently correlates with the tumor inflammation signature [35]. This further strengthens current approaches to combine anti-CD38 with checkpoint inhibitor therapy [36]. Primary EAC, presenting a high inflammation signature in combination with dominant CD8 and CD38 expression might be promising targets for such a combinatorial treatment.

Recently, radiochemotherapy was thought to increase the presence of neoantigens as a result of its mutagenic character [37]. In general, a greater overall survival as well as higher efficiency of immunotherapy with checkpoint inhibitors are associated with higher neoantigen burden and CD8 + T cell infiltration [38]. Nevertheless, we identified that even the presence of cancer testis antigens is significantly decreased after radiochemotherapy in esophageal carcinoma. This is in concordance with another study conducted in ovarian cancer, where the authors found the predicted increase in neoantigens to be due to pre-existing mutational processes rather than from mutagenesis induced by chemotherapy [38].

The present study demonstrated some important new findings: (a) the influence of the currently used neoadjuvant treatment, (b) the unexpected higher expression of checkpoint markers like LAG3, TIM-3, CTLA4 and CD276 in comparison to PD-L1/PD-1 supporting clinical trials analyzing the efficacy of a combination of different checkpoint inhibitors in EACs, (c) the importance of immune escape mechanism like a high CD38 or LILRB1 expression in EACs.

TIM-3, also known as HAVCR2 could be an interesting and promising target for anticancer immunotherapy, since it is expressed on a variety of T-cells, DCs (dendritic cells), macrophages and monocytes and elicits a strong innate anti-tumor immune response. A variety of different studies have proven comparable effects of anti TIM-3 inhibition [39]. PD-1, TIM-3 and LAG-3 inhibitors are able to enhance the T-cell response to tumor antigens. Moreover a synergistic function of the above mentioned could enhance the response in combinatorial therapies [40, 41]. LAG-3 as a further promising immune-checkpoint has been investigated in various clinical trials and combinatorial treatment with anti-PD1 therapy showed high efficacy especially in PD1 resistant settings [40].

An increased expression of CD38 is correlated with a poor prognosis in chronic lymphocytic leukemia cells. Administration of the anti-CD38 mAb daratumumab has been shown to induce apoptosis and promotion of immune-initiated clearance [42]. A combinatorial screening of PD1/PD-L1 and CD38 could be of interest for diagnostics to predict response to PD-L1 blockade or even allow for a combinatorial treatment with checkpoint inhibitors and CD38 blocking agents to improve patients’ outcome.

Furthermore to further identify prognostic markers, a clinical follow up of patients with different immunoprofiles could be of high interest. Additionally to the relative low sample size within the cohort of NACT-treated EACs, it has to be kept in mind that this group is heterogeneous according to the type of treatment regimen. To strengthen the findings described within this study, a larger and homogeneous cohort of NACT-trated EAC patients could be tested in future research. Although heterogeneity of this sub-chohort, the herein described major influences of treatment to the immune profile are similar regardless of treatment regimen. There is a difference between FLOT and CROSS treated patients on gene expression as described in Fig. 5, but the major influence of NACT treatment (a down-regulation of nearly all important checkpoint markers and inflammatory related genes in the local microenvironment) is consistent between both subgroups.

## Electronic supplementary material

Below is the link to the electronic supplementary material.

Supplementary file1 (PDF 583 kb)

## Abbreviations

ARG1: Arginase 1
CTA: Cancer testis antigen
CCR3: C–C chemokine receptor type 3
CXCL9: Chemokine (C–X–C motif) ligand 9
CD: Cluster of differentiation
CROSS: CROSS-trial regimen with paclitaxel, carboplatin and 41.4 Gy/23 fractions
CSM: Consensus molecular subtype
CTLA-4: Cytotoxic T-lymphocyte-associated protein 4
DNAse: Deoxyribonuclease
EBV: Epstein–Barr virus
EAC: Esophageal adenocarcinoma
FC: Fold change
FFPE: Formalin-fixed and paraffin embedded
FLOT: FLOT-trial regimen with 5FU, Folinic acid, Oxaliplatin, Docetaxel
H&E: Hematoxylin–eosin
HAVCR2: Hepatitis A virus cellular receptor 2
HER2: Human epidermal growth factor receptor 2
HLA: Human histocompatibility leukocyte Ag
IDO: Indoleamine-pyrrole 2,3-dioxygenase
IFN-γ: Interferon-gamma
LILRB1: Leukocyte immunoglobulin-like receptor subfamily B member 1
LAG3: Lymphocyte-activation gene 3
MHC: Major histocompatibility complex
mRNA: Micro ribonucleic acid
MSI: Microsatellite instability
mAb: Monoclonal Ab
NACT: Neoadjuvant chemotherapy
NSCLC: Non-small-cell lung carcinoma
OS: Overall survival
PD-1: Programmed death 1 receptor
PD-L1: Programmed death ligand 1
RCT: Radio chemotherapy
RNA: Ribonucleid acid
TIM3: T-cell immunoglobulin and mucin-domain containing-3
TGF: Transforming growth factor
TIL: Tumor infiltrating lymphocyte
TIS: Tumor inflammation signature
TMB: Tumor mutation burden
TNF: Tumor necrosis factor

## References

[1] Edgren G (2013). A global assessment of the oesophageal adenocarcinoma epidemic. Gut.

[2] Lagergren J, Lagergren P (2013). Recent developments in esophageal adenocarcinoma. CA Cancer J Clin.

[3] Maret-Ouda J, El-Serag HB, Lagergren J (2016). Opportunities for preventing esophageal adenocarcinoma. Cancer Prev Res.

[4] Gavin AT (2012). Oesophageal cancer survival in Europe: a EUROCARE-4 study. Cancer Epidemiol.

[5] Njei B, McCarty TR, Birk JW (2016). Trends in esophageal cancer survival in United States adults from 1973 to 2009: a SEER database analysis. J Gastroenterol Hepatol.

[6] Kapoor H, Agrawal DK, Mittal SK (2015). Barrett's esophagus: recent insights into pathogenesis and cellular ontogeny. Transl Res.

[7] Olson BM, McNeel DG (2012). Antigen loss and tumor-mediated immunosuppression facilitate tumor recurrence. Expert Rev Vaccines.

[8] Dyck L, Mills KHG (2017). Immune checkpoints and their inhibition in cancer and infectious diseases. Eur J Immunol.

[9] Raufi AG, Klempner SJ (2015). Immunotherapy for advanced gastric and esophageal cancer: preclinical rationale and ongoing clinical investigations. J Gastrointest Oncol.

[10] Ferris RL (2016). Nivolumab for recurrent squamous-cell carcinoma of the head and neck. N Engl J Med.

[11] Borghaei H (2015). Nivolumab versus docetaxel in advanced nonsquamous non–small-cell lung cancer. N Engl J Med.

[12] Motzer RJ (2015). Nivolumab versus everolimus in advanced renal-cell carcinoma. N Engl J Med.

[13] Larkin J (2015). Combined nivolumab and ipilimumab or monotherapy in untreated melanoma. N Engl J Med.

[14] Bockorny B, Pectasides E (2016). The emerging role of immunotherapy in gastric and esophageal adenocarcinoma. Future Oncol.

[15] Vrana D, Matzenauer M, Melichar B (2017). Current status of checkpoint inhibitors in the treatment of esophageal and gastric tumors - overview of studies. Klin Onkol.

[16] Gjerstorff MF, Andersen MH, Ditzel HJ (2015). Oncogenic cancer/testis antigens: prime candidates for immunotherapy. Oncotarget.

[17] Ayers M (2017). IFN-gamma-related mRNA profile predicts clinical response to PD-1 blockade. J Clin Invest.

[18] Holscher AH (2007). Laparoscopic ischemic conditioning of the stomach for esophageal replacement. Ann Surg.

[19] Danaher P (2018). Pan-cancer adaptive immune resistance as defined by the tumor inflammation signature (TIS): results from the cancer genome atlas (TCGA). J ImmunoTherapy Cancer.

[20] Merck, Merck’s KEYTRUDA® (pembrolizumab) significantly improved overall survival (OS) compared to chemotherapy in patients with advanced esophageal or esophagogastric junction carcinoma whose tumors express PD-L1 (CPS ≥10). 2018.

[21] Hewitt LC (2018). Epstein–Barr virus and mismatch repair deficiency status differ between oesophageal and gastric cancer: a large multi-centre study. Eur J Cancer.

[22] Cancer Genome Atlas Research N et al. (2017) Integrated genomic characterization of oesophageal carcinoma. Nature 541(7636), 169–175. 10.1038/nature20805 PMC5651175 28052061

[23] Roelands J et al (2017) Immunogenomic classification of colorectal cancer and therapeutic implications. Int J Mol Sci 18(10) 10.3390/ijms18102229 PMC5666908 29064420

[24] Tang H, Qiao J, Fu Y-X (2016). Immunotherapy and tumor microenvironment. Cancer Lett.

[25] van Meir H (2016). Impact of (chemo)radiotherapy on immune cell composition and function in cervical cancer patients. Oncoimmunology.

[26] Jarosch A (2018). Neoadjuvant radiochemotherapy decreases the total amount of tumor infiltrating lymphocytes, but increases the number of CD8+/Granzyme B+ (GrzB) cytotoxic T-cells in rectal cancer. Oncoimmunology.

[27] Shen X, Zhao B (2018) Efficacy of PD-1 or PD-L1 inhibitors and PD-L1 expression status in cancer: meta-analysis. BMJ 362 10.1136/bmj.k3529 PMC6129950 30201790

[28] Castellanos JR (2017). B7–H3 role in the immune landscape of cancer. Am J Clin Exp Immunol.

[29] Lin C-F (2017). Escape from IFN-γ-dependent immunosurveillance in tumorigenesis. J Biomed Sci.

[30] Beatty GL, Gladney WL (2015). Immune escape mechanisms as a guide for cancer immunotherapy. Clin Cancer Res.

[31] Rodriguez JA (2017). HLA-mediated tumor escape mechanisms that may impair immunotherapy clinical outcomes via T-cell activation. Oncol Lett.

[32] Campoli M, Chang CC, Ferrone S (2002). HLA class I antigen loss, tumor immune escape and immune selection. Vaccine.

[33] Barkal AA (2018). Engagement of MHC class I by the inhibitory receptor LILRB1 suppresses macrophages and is a target of cancer immunotherapy. Nat Immunol.

[34] Gajewski TF (2013). Cancer immunotherapy strategies based on overcoming barriers within the tumor microenvironment. Curr Opin Immunol.

[35] Chen L (2018). CD38-mediated immunosuppression as a mechanism of tumor cell escape from PD-1/PD-L1 blockade. Cancer Discov.

[36] Chen L (2018). Targeting CD38 to improve anti-PD-1/CTLA-4 combination therapy in lung cancer. J Clin Oncol.

[37] Brown JS, Sundar R, Lopez J (2018). Combining DNA damaging therapeutics with immunotherapy: more haste, less speed. Br J Cancer.

[38] O'Donnell T (2018). Chemotherapy weakly contributes to predicted neoantigen expression in ovarian cancer. BMC Cancer.

[39] He Y (2018). TIM-3, a promising target for cancer immunotherapy. OncoTargets Therapy.

[40] Long L (2018). The promising immune checkpoint LAG-3: from tumor microenvironment to cancer immunotherapy. Genes Cancer.

[41] Marcq E (2017). Abundant expression of TIM-3, LAG-3, PD-1 and PD-L1 as immunotherapy checkpoint targets in effusions of mesothelioma patients. Oncotarget.

[42] Manna A (2018). Using anti-CD38 immunotherapy to enhance anti-tumor T-cell immunity in chronic lymphocytic leukemia (CLL). J Immunol.

