# Supplementary material for: Immune profile and immunosurveillance in treatment-naive and neoadjuvantly treated esophageal adenocarcinoma
Source: Cancer Immunol Immunother. 2020 Jan 20;69(4):523–33. doi: 10.1007/s00262-019-02475-w (PMC7113210; doi:10.1007/s00262-019-02475-w)
Supplement: Supplementary file 1 — Supplementary file1 (PDF 583 kb) [file 262_2019_2475_MOESM1_ESM.pdf]

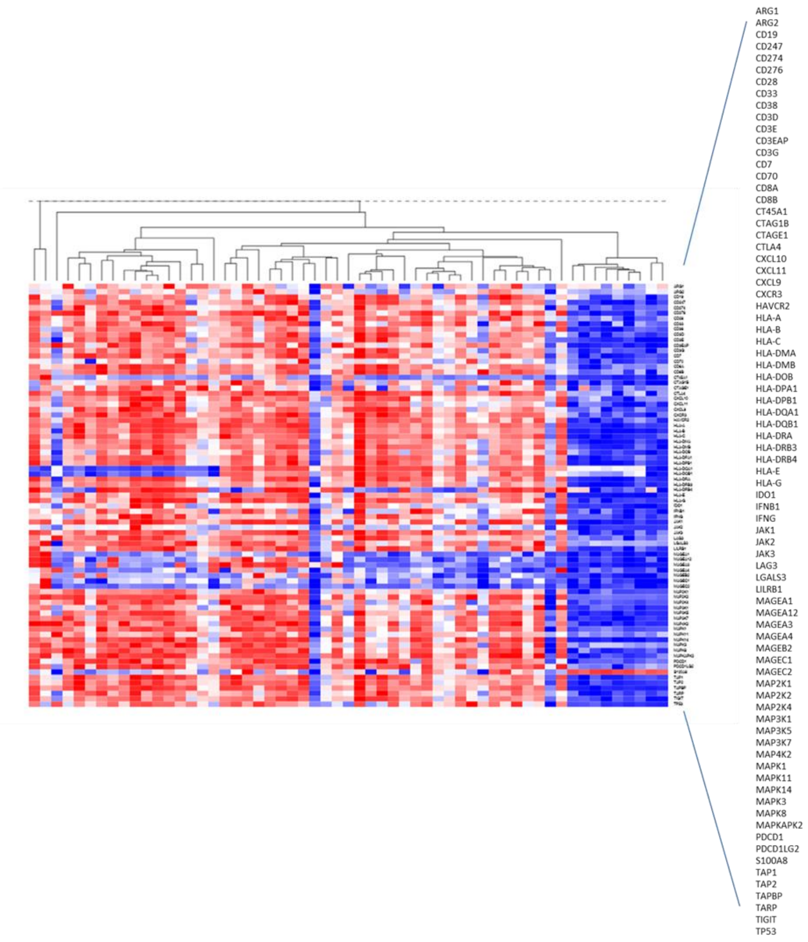

Sup Fig1: Heatmap.showing differential expression of immune profile- and immune escape related genes. Levels of downregulated expression (blue) or upregulated expression (red) are shown on a log2 scale from low to high expression of each gene.

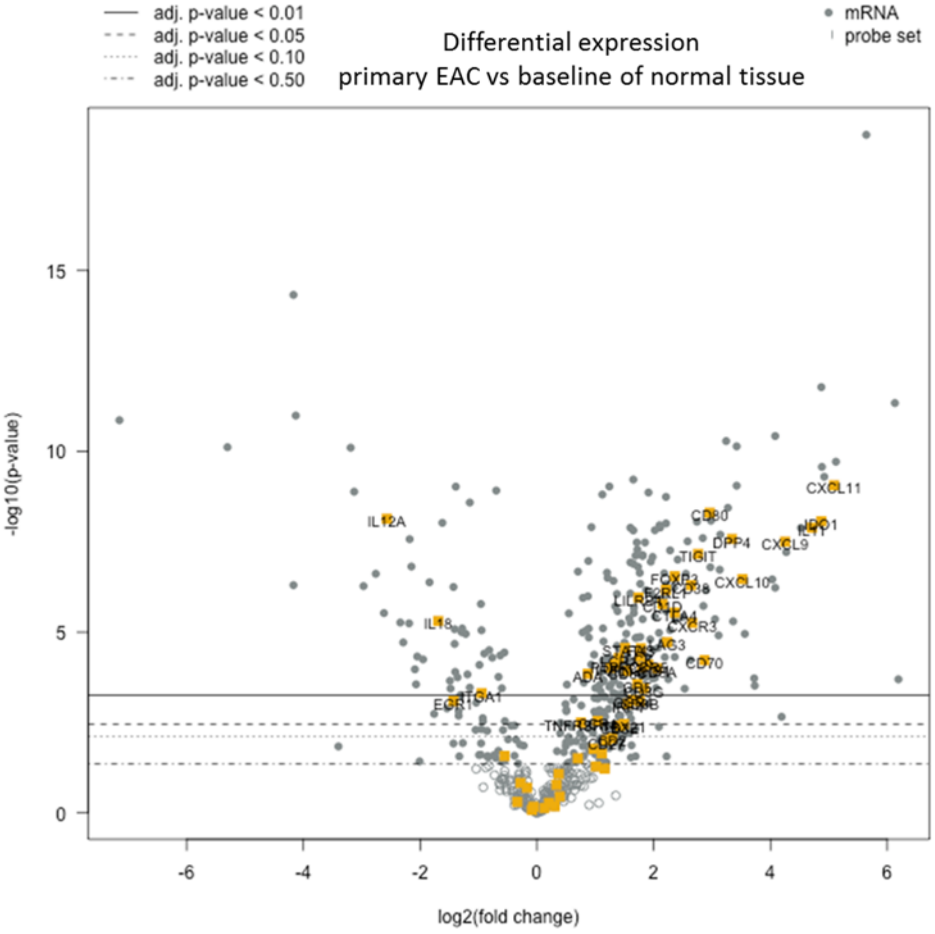

Sup Fig2: Differential expression of genes associated with T cell function. Expression of immune-related genes in primary EAC. Fold change expression is shown as log2 values. Level of significance (p-value) is represented as –log10 on the y-axis, thresholds of significants are represented by different line, as indicated on top. Yellow dots indicate genes related to T cell function.

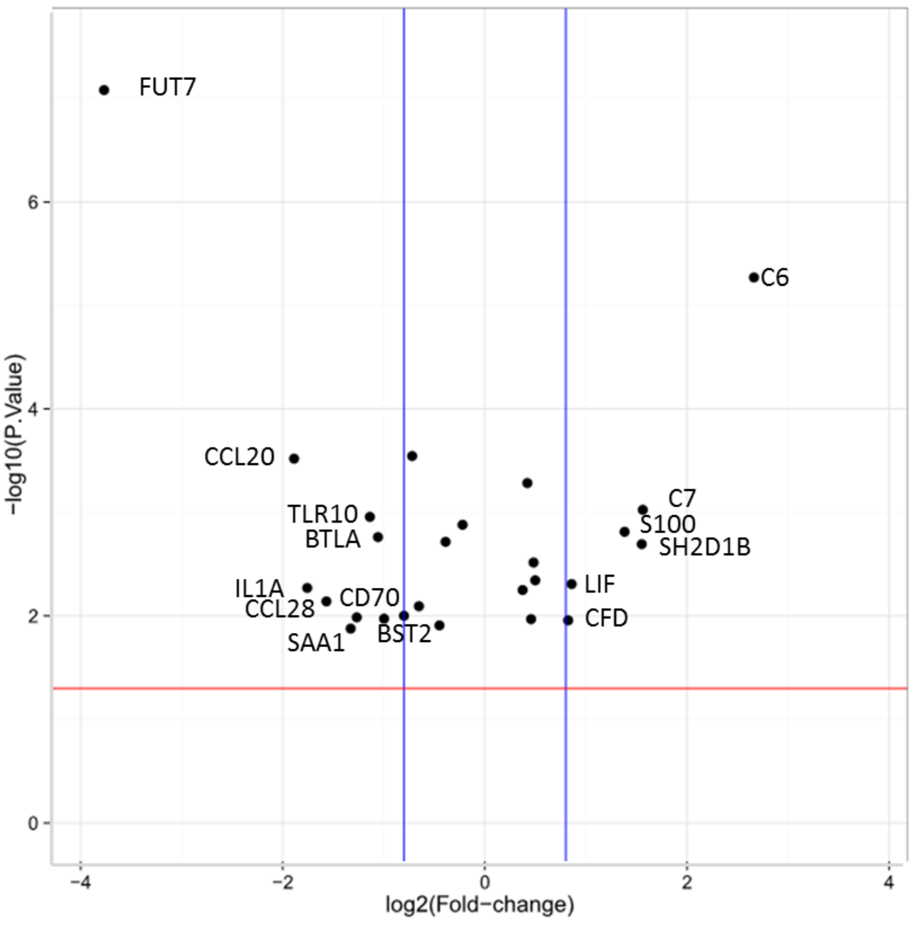

Sup Fig3: TOP 25 genes of post NACT versus naive EAC. Volcano plot for log2 foldchanges in mRNA gene expression levels in post NACT vs primary resected treatment- naive EAC. Thresholds of significance p-value:1.3 ; log2FC: 0.8

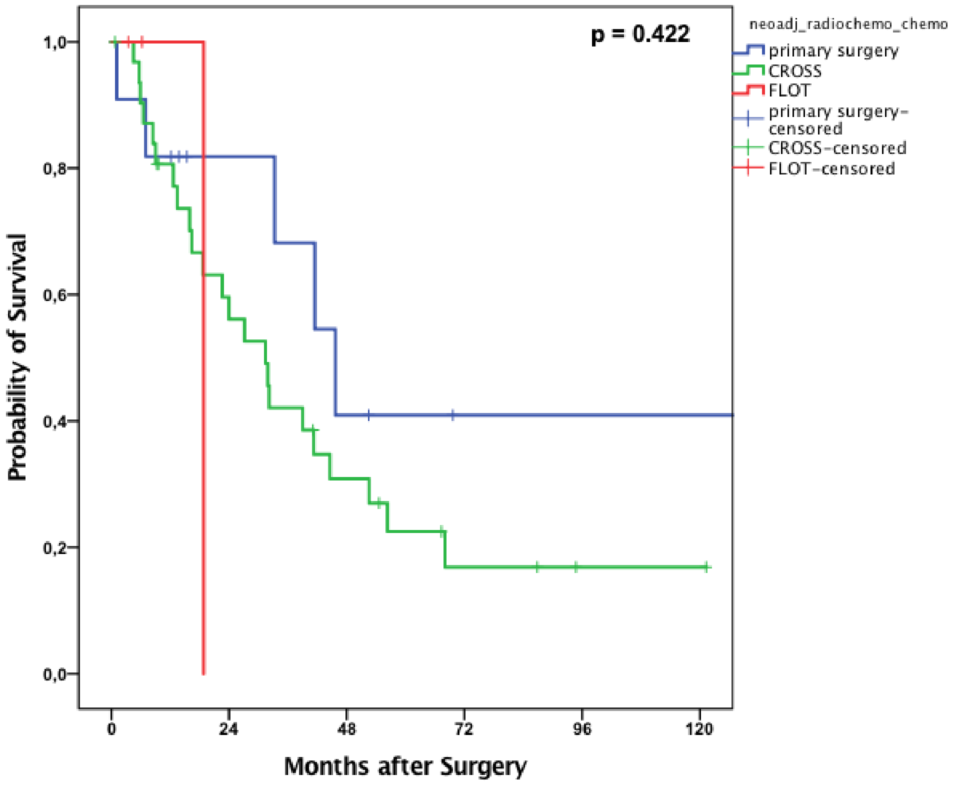

Sup Fig4: Patients survival data depicted in a Kaplan-Meyer graph for primary resected (blue) and NACT-treated patients (green: CROSS, red: FLOT)
